# Genetic Risk Score Predictive of the Plasma Triglyceride Response to an Omega-3 Fatty Acid Supplementation in a Mexican Population

**DOI:** 10.3390/nu11040737

**Published:** 2019-03-29

**Authors:** Bastien Vallée Marcotte, Frédéric Guénard, Julien Marquis, Aline Charpagne, Felipe Vadillo-Ortega, Maria Elizabeth Tejero, Aristea Binia, Marie-Claude Vohl

**Affiliations:** 1 Institute of Nutrition and Functional Foods (INAF), Laval University, 2440 Hochelaga Blvd, Quebec, QC G1V 0A6, Canada; bastien.vallee-marcotte.1@ulaval.ca (B.V.M.); frederic.guenard@fsaa.ulaval.ca (F.G.); 2 Nestlé Institute of Health Sciences, Nestlé Research, Vers-chez-les-Blanc, P.O. Box 44, 1000 Lausanne 26, Switzerland; Julien.marquis.1@unil.ch (J.M.); ACharpagne@sophiagenetics.com (A.C.); aristea.binia@rdls.nestle.com (A.B.); 3 Unidad de Vinculación de la Facultad de Medicina UNAM en el Instituto Nacional de Medicina Genómica, Ciudad de México 14610, Mexico; fvadillo@inmegen.gob.mx; 4 Laboratorio de Nutrigenética y Nutrigenómica Instituto Nacional de Medicina Genómica, Ciudad de México 14610, Mexico; etejero@inmegen.gob.mc

**Keywords:** genetic risk score, omega-3 fatty acids, triglycerides, nutrigenetics

## Abstract

Our group built a genetic risk score (GRS) of the plasma triglyceride (TG) response to an omega-3 (n-3) fatty acid (FA) supplementation in Caucasian Canadians that explained 21.53% of the TG variance. The objective was to refine the GRS by fine mapping and to test its association with the TG response in young Mexican adults. A total of 191 participants underwent a 6-week n-3 FA supplementation providing 2.7g/day of docosahexaenoic and eicosapentaenoic acids. Using quantitative polymerase chain reaction (PCR), 103 single-nucleotide polymorphisms (SNPs) were genotyped. A stepwise regression adjusted for age, sex, and body mass index (BMI) was used to select the strongest SNPs to include in the genetic risk model. A GRS was calculated from the sum of at-risk alleles. The contribution of the GRS to the TG response was assessed by ANCOVA with age, sex, and BMI included in the model. Several differences in allele frequency were observed between Canadians and Mexicans. Five lead SNPs were included in the genetic risk model, in which the GRS accounted for 11.01% of the variance of the TG response (*p* < 0.0001). These findings highlight the important contribution of genetic factors to the heterogeneity of the TG response to an n-3 FA supplementation among Mexicans.

## 1. Introduction

The metabolic response to a treatment or a dietary intervention, even when proven effective, can vary considerably from one individual to another, sometimes resulting in an even more deteriorated profile after the treatment or the intervention [1,2,3,4,5]. In this sense, numerous studies have demonstrated that the metabolic response to an omega-3 (n-3) fatty acid (FA) supplementation, particularly at pharmacological doses, is highly heterogeneous, and so is the plasma triglyceride (TG) response [6,7].

Genetic factors have been reported to contribute significantly to this inter-individual variability in the response, often in studies using a hypothesis-driven approach [6,8,9,10,11,12,13,14]. Conversely, our group recently used a hypothesis-free approach to identify novel genetic determinants of the plasma TG response to an n-3 FA supplementation by conducting a genome-wide association study (GWAS) in a sample of French Canadians from the province of Quebec (Canada) in the Fatty Acid Sensor (FAS) Study [15]. A genetic risk score (GRS) was computed by summing the number of alleles from GWAS hits and explained 21.53% of the variation in TG response [15]. Results have been replicated in participants of the European FINGEN Study [15].

GRSs have been proven to be an effective tool for predicting the response to such interventions [16,17,18]. However, to date, most studies investigating the heterogeneity of the contribution of genetic factors to the plasma TG response to an n-3 FA supplementation were conducted in Caucasian populations [6,19,20,21]. Since allele frequency can considerably vary between populations, the predictive capacity of a GRS may not be generalizable to other ethnic groups. Replication of GRS findings is therefore necessary to provide more robust evidence regarding their efficacy and generalizability in various ethnic groups.

A previous intervention study in which young Mexican adults were supplemented with 2.7 g of n-3 FA per day showed that genetic variation in the peroxisome proliferator-activated receptors α and γ2 (*PPARα* and *γ2*) genes moderately influence the triglyceride response to the intervention [22]. The objective of the present study was to test the association of a GRS predictive of the plasma TG response to the n-3 FA supplementation in this study population and to refine it via fine mapping of GWAS-associated loci. 

## 2. Materials and Methods 

### 2.1. Population

The study population, intervention and genotyping procedures were detailed in a previous publication [22]. Briefly, inclusion criteria were: aged between 18 and 40 years old, BMI between 18.5 and <30 kg/m^2^, no ongoing medication, no vitamin nor lipid supplements prior to or during the intervention, sedentary to moderate level of physical activity according to the IPAQ questionnaire [23]. Exclusion criteria were: active smoking, excessive alcohol consumption, illness two weeks prior to the intervention, any condition requiring medical treatment during the study, participation to another clinical trial four weeks before the intervention. A total of 191 participants who completed the intervention had available data for statistical analyses.

### 2.2. Intervention

The intervention was conducted at Universidad Iberoamericana and Universidad Nacional Autonoma de Mexico (UNAM) in Mexico City between November 2013 and May 2014. The intervention consisted of a 6-week n-3 FA supplementation of fish oil (GNC Preventive Nutrition^®^ Triple Strength Fish Oil) comprising three visits. At the first visit, anthropometric measurements and blood samples were taken. Participants also had a dietary evaluation using a validated food frequency questionnaire (SNUT) [24]. They were also given the necessary capsules for first three weeks. Each capsule contained 253 mg of docosahexaenoic acid (DHA) and 647 mg of eicosapentaenoic acid (EPA), for a total of 900 mg of DHA/EPA per capsule. Participants had to take three capsules a day, providing 2.7 g/day of DHA/EPA. Participants were asked to consume the capsules with food to optimize the FA absorption [25]. At the second visit, participants received clinical and biochemical results and had nutrition follow-up. They were then given the capsules for the remaining three weeks. At the third visit, the same parameters as baseline were re-evaluated. A 24-h food recall questionnaire, physical activity and consumption of medication and/or supplementation questionnaires were administered at each visit. Compliance was assessed by the returning of remaining capsules at each visit and FA incorporation in red blood cells’ membranes. A final number of 191 participants completed the intervention and had biochemical data usable for statistical analysis.

### 2.3. Single-Nucleotide Polymorphisms Selection for Genotyping

Single-nucleotide polymorphisms (SNPs) were selected according to results previously published by our research group on the FAS Study [15,26]. First, SNPs that were identified in the GWAS of the FAS Study were selected for genotyping in a way to build the GRS in the Mexican population using the same SNPs [15]. A total of 12 SNPs from Rudkowska et al. were selected and submitted for genotyping [15]. SNPs were located in the following GWAS loci: *IQCJ-SCHIP1*, *NXPH1*, *PHF17*, *MYB*, *NELL1* and *SLIT2*.

In order to produce a refined GRS with more markers, candidate SNPs that were shown to modulate plasma TG levels and the TG response following the n-3 FA supplementation (gene-diet interactions) in another study by our group were also selected [26]. Briefly, the Haploview software v4.2 for SNPs selection along with quantitative polymerase chain reaction (PCR) for genotyping were jointly used to increase the density of GWAS hits in order to identify additional SNPs associated with the plasma TG response to an n-3 FA supplementation. A total of 87 SNPs in the same GWAS loci (except for *NELL1* and *SLIT2*, for which genotyping was not conducted) were selected and genotyped. Several other SNPs located in genes known to be associated with the TG trait were also added. The final list included SNPs located on the *salt-inducible kinase 3* (*SIK3*), *lipoprotein lipase* (*LPL*) and the *MLX Interacting Protein Like* (*MLXIPL*) genes [27,28,29]. A final number of 103 SNPs were kept for statistical analysis.

### 2.4. Genotyping

Trizol reagent (Thermo Fisher Scientific, Eculbens, Switzerland) was used to extract DNA from mononuclear cells according to manufacturer’s instructions. Quality of isolated DNA was evaluated in a Nanodrop Spectrophotometer (Thermo Fisher Scientific) and agarose gel electrophoresis stained with ethidium bromide. A subsample was evaluated in an Agilent 250 bioanalyzer (Agilent Palo Alto, CA, USA). All samples met quality control requirements. 

All samples were quantified by a fluorimetric method (Picogreen, Thermo Fisher Scientific). All 103 SNPs but seven (see below) were assayed using SNP Type Assays from Fluidigm following manufacturer’s recommendations. Briefly, 12.5 ng genomic DNA was pre-amplified for 14 cycles, diluted 100-fold in low TE buffer (Thermo Fisher Scientific), loaded into a 96 × 96 Dynamic Array IFC (Fluidigm, Les Ulis, France) with individual SNP Type assays, and run on a Biomark HD (Fluidigm). Individual genotypes were manually reviewed using the SNP Genotyping Analysis software (version 4.1.2, Fluidigm). Control samples of known genotypes were included in the overall procedure to facilitate genotype clusters identification. The seven remaining SNP were assayed using pre-designed Taqman assays (Thermo Fisher Scientific) run from 20 ng DNA into 10 uL reactions using the LightCycler^®^ 1536 DNA Probes Master mix (Roche, Risch-Rotkreuz, Switzerland) on a Light Cycler 480 II (Roche) equipped with a 384 wells block. Analysis was performed with the LightCycler^®^ Software (release 1.5.0, Roche), again taking advantage of control samples of known genotypes.

### 2.5. SNP and Statistical Analysis

Participants’ characteristics (mean values ± standard deviation of anthropometric and biochemical parameters) at baseline (pre-) and post-intervention were calculated for both responders and non-responders to the n-3 FA supplementation. Responders/non-responders were defined as follow: Participants with a delta (Δ) TG <0 were considered responders to the n-3 FA supplementation whereas participants with a ΔTG ≥0 were considered non-responders. Values pre- vs post-intervention were compared within and between the two subgroups using a *t*-test. 

Hardy–Weinberg equilibrium was evaluated using a Chi-squared test. Minor allele frequency between responders and non-responders was calculated and compared between the two subgroups using PLINK software. Proportions of non-responders and responders carrying the minor allele of a SNP were compared. For SNPs that were genotyped in participants of both cohorts (French Canadians and Mexicans), allele frequency distribution between participants of the two samples was compared using a Chi-squared test.

A GRS was computed by summing the number of risk alleles of each Mexican participant using all 103 SNPs. To do so, minor alleles with an odds ratio >1 were attributed a +1 value and minor alleles with an odds ratio <1 were attributed a -1 value. Major alleles had a value of 0. The contribution of the GRS to the TG response (delta TG) was assessed by ANCOVA with age, sex and BMI included in the model. Significance was set at *p* < 0.05. Statistical analyses were conducted in SAS statistical software v9.4.

This study was approved by the Ethics Committees at Instituto Nacional de Medicina Genomica (INMEGEN), Western Institutional Review Board and Universidad Nacional Autonoma de Mexico (UNAM). Informed consent was reviewed and signed by all participants before data collection. The study was registered in www.clinical.trials.gov as NCT02296385.

## 3. Results

Fully detailed characteristics of participants have been previously published [22]. Anthropometric measurements and TG levels of responders and non-responders pre- and post-supplementation are presented in Table 1. The mean BMI of participants was within normal range, although a minority of participants were slightly overweight. Mean weight of responders and non-responders stayed stable throughout the supplementation protocol. Plasma TG levels significantly changed during the supplementation for both subgroups as expected. A proportion of 40.8% of the Mexican population was non-responsive to the n-3 FA supplementation, as opposed to 59.2% of responders. Non-responders had baseline TG levels lower than responders, as previously observed in the FAS Study [15].

Among the 103 SNPs tested in the Mexican population, four were not in Hardy Weinberg Equilibrium (rs11769942, rs7793115, rs4141002, rs17150341). Minor allele frequency comparison between Canadian Caucasian and Mexican populations is presented in Table 2 [26]. For the majority of SNPs, minor allele frequency was different between the two cohorts. 

To replicate the GRS previously computed from GWAS hits in the FAS Study, a first GRS was here calculated using as many markers previously used in the FAS study GRS as possible [15]. In the FAS study GRS, a total of 10 SNPs were used. Because several SNPs were either in linkage disequilibrium (*r*^2^ > 0.8) or not designable in the Mexican cohort, a total of seven genotyped SNPs were used to calculate the GRS. In an ANCOVA including age, sex and BMI in the model, the GRS did not significantly explain TG variation during the supplementation (*p* = 0.98) (data not shown).

**Figure 1:**
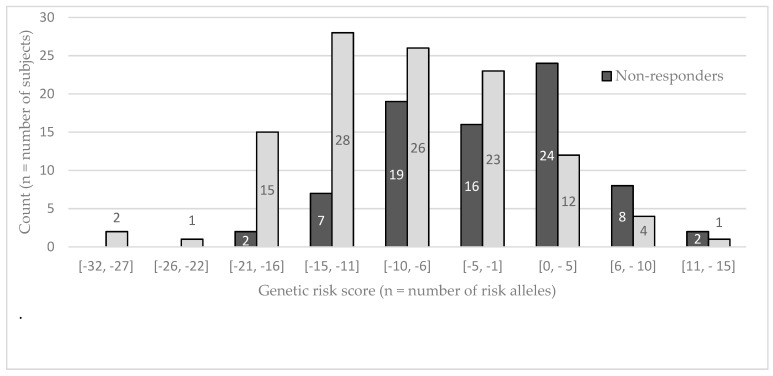
Risk score (GRS) distribution in the Mexican population according to 103 SNPs (*n* = 191 individuals). If a GRS is positive, the subject carries more at-risk alleles. If a GRS is negative, the subject carries more beneficial alleles.

**Figure 2:**
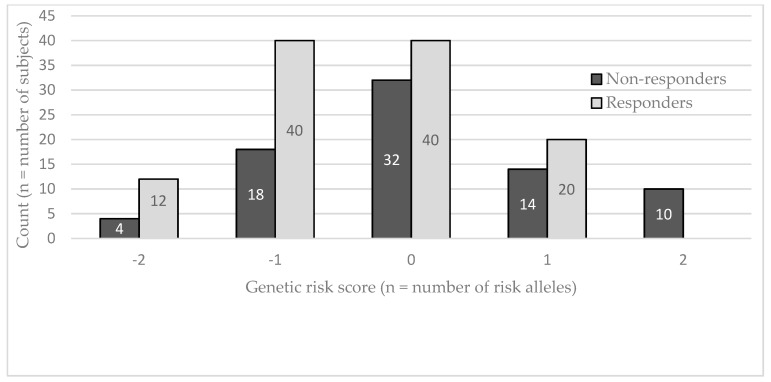
Risk score (GRS) distribution in Mexicans according to 5 SNPs (*n* = 190 individuals). If a GRS is positive, the subject carries more at-risk alleles. If a GRS is negative, the subject carries more beneficial alleles.

A second GRS was then computed using all genotyped SNPs in the Mexican population. Figure 1 shows the GRS distribution in the study population using all 103 SNPs. A higher score means the subject carries more at-risk alleles, as opposed to a lower score that means the subject carries more beneficial alleles. In a general linear model adjusted for age, sex and BMI, the GRS explained 4.37% of TG variation (*p* = 0.0038). A stepwise regression for bidirectional elimination adjusted for age, sex and BMI was used to select the most relevant SNPs to include in the general linear model (GLM). The procedure left five SNPs (*NXPH1* rs10265408, rs10486228, rs17150341, rs6974252, and *IQCJ-SCHIP1* rs2595241) to be included in the general linear model. A genetic risk model of these five SNPs (5-SNPs GRS) explained 11.01% of the TG variation (*p* < 0.0001). Figure 2 shows the GRS distribution in the study population using these five SNPs (5-SNPs GRS). A flowchart summarizing the genetic risk score development is presented as supplementary material.

It was also verified whether computing a 5-SNPs GRS only using participants with the most extreme responses to the n-3 FA supplementation, that is participants having the greatest ΔTG, negative or positive, would improve the percent explained by the GRS in the general linear model. Because the FAS GWAS (and therefore the FAS GRS) was computed using the 141 most responsive subjects, that is all non-responders (*n* = 60) and the greatest responders (*n* = 81), the 5-SNPs GRS was recalculated after eliminating participants with lower ΔTG in a way to reach a responders: non-responders ratio of 50:50. Table 3 presents differences in the TG variation explained by the 5-SNPs GRS in the general linear model with participants retrenched. As participants were subtracted, the percentage of TG variance explained by the 5-SNPs GRS increased to a maximum of 29.10% with 56 participants left (*p* < 0.0001). Taking more participants out of the calculation resulted in a decrease in the percentage explained. 

## 4. Discussion

The aim of this study was to verify whether a GRS of the plasma TG response to an n-3 FA supplementation developed within a French Canadian sample can explain the plasma TG response to n-3 FA in Mexicans. In this study, genetic risk models were built first by including genotyped SNPs and secondly by narrowing down the number of SNPs to the most relevant ones. To our knowledge, this is the first study to fully explore the contribution of genetic factors to the response of plasma TG levels to an n-3 FA supplementation in Mexicans, by combining the effect of several SNPs associated with the TG response. According to previous studies, dyslipidemias with increased concentration of TG affect at least one third of the adult population in Mexico in association with the combined prevalence of overweight and obesity, affecting 72.5% of adults ≥20 years old [30,31,32]. 

It was first observed that the proportion of responders vs non-responders is different between the two populations. In Mexicans, 40.8% of participants were non-responsive. This proportion of non-responders is higher than what has been observed in the FAS Study, where non-responders corresponded to 28.8% of the population. A similar proportion of non-responders (~30%) was reported in the European FINGEN study also conducted on Caucasians [7,14,15]. In addition, differences in allele frequency between the two populations were detected. These first observations suggest substantial differences in the global genetic makeup, as expected, in French Canadians and Mexicans.

In the FAS Study, the GRS was computed out of 10 SNPs on the 141 most responsive subjects to the n-3 FA supplementation and accounted for 21.53% of the variation in TG response [15]. In the present study, a 7-SNPs GRS was built in order to replicate as similarly as possible the FAS Study GRS (10 SNPs). This GRS did not explain TG variation. This discrepancy can first be explained by the above-mentioned differences in genetic makeup between Canadians and Mexicans. Another explanation may be the use of seven SNPs instead of 10 to build the GRS. Refined genetic risk model with 103 SNPs significantly contributed to TG variation, and selecting the five most dominant SNPs that were driving the associations between TG levels and SNPs in the Mexican population resulted in a considerably improved model. Also, a 5-SNPs GRS computed in the Mexican population with 141 most responsive subjects would account for about 13% of the TG variation, which is lower than the percent of the TG variation explained by the GRS in the FAS Study. Our findings show that the GRS accounts for a larger proportion of the TG variance when participants with the lowest magnitude of TG response are excluded from the calculation. This trend could be explained by the possibility that participants who are the most sensitive to n-3 FA supplementation (positively and negatively) are the ones who carry the most beneficial (responders) or detrimental (non-responders) alleles. 

Altogether, results of the present study clearly confirm the implications of genomic regions previously identify by GWAS in the FAS Study, and demonstrate the potential of fine mapping to refine predictive models. These observations also show that despite a great predictive capacity, its predictive capacity slightly differs between ethnic groups. Still, the proportion of the TG variance explained by the GRS in the Mexican population (11.01%) is considerably high in comparison to other similar GRS of the TG trait. A recently published weighted GRS of TG levels was constructed from 40 SNPs previously associated with TG levels [33]. The study population was composed of American women only who participated in the Women’s Genome Health Study (*n* = 21840) [33]. Similarly to the present findings, the genetic risk model explained 4.99% of the TG variance [33]. Moreover, the addition of each TG risk allele was significantly associated with a 1.01% increase in TG levels (*p* < 0.0001) [33]. A GRS of TG levels was recently built in a cohort of Filipino women (*n* = 1649) from nine SNPs previously associated with TG levels [34]. Consistently with the present study, a significant proportion of the variance of TG levels was explained by the GRS alone (6% of log TG levels) [34]. The addition of each TG risk allele increased by approximately 7% TG levels (β = 0.07, 95% Confidence Interval (CI) 0.06–0.08, *p* = 3.38 × 10^−28^) [34]. Two SNPs that were used in the genetic risk calculation, rs2286276 (*TBL2-MLXIPL*) and rs964184 (*APOC3*), were also used in the genetic risk calculation of the present study [34]. Another GRS of TG levels was constructed with participants of The Cardiovascular Risk in Young Finn Study, a Finnish population-based prospective cohort study, from 24 TG-associated SNPs [35]. Again, the GRS significantly contributed to TG levels [35]. Subjects in the low and medium GRS groups showed average baseline TG levels approximately 20% (*p* = 0.0001) and 10% (*p* = 1.0 × 10^−4^) lower in comparison to participants in the high GRS group. Moreover, León-Mimila et al. computed a GRS of hepatic TG content from four hits of a GWAS of non-alcoholic fatty liver disease [36]. Similarly to the present study, the GRS was computed in a population of 130 Mexicans Mestizo, but with morbid obesity, and participants also had bariatric surgery [36]. A significant stepwise increase in hepatic TG content was observed as risk alleles were added (*p* = 1.0 × 10^−4^) [36]. The GRS was also associated with hepatic TG content (*p* = 0.048) [36]. Despite the clear important contribution of gene variations to the TG trait in many populations of different ethnic backgrounds, the four GRS discussed above are predictive of the plasma TG levels only and are thus independent of the effect of n-3 FA on plasma TG levels.

## 5. Conclusions

In conclusion, the present replication study is the first to demonstrate that the applicability of the GRS of the plasma TG response to n-3 FA, previously built in Caucasians from the FAS Study, can be refined and then extended to Mexican populations. Results of the present study could be of important contribution in the treatment of hypertriglyceridemia by identifying patients who are most likely to respond effectively to an n-3 FA supplementation and could ultimately concretize the application of personalized dietary recommendations to patients based on their genetic profile in clinical practice. Further research should focus on further investigating the genetic contribution to the heterogeneity in the response to an n-3 FA supplementation in different ethnic groups.

## Figures and Tables

**Table 1:** Characteristics of Mexicans at baseline and post-intervention (*n* = 191 individuals).

Characteristics	Responders (*n* = 113) *			Non-Responders (*n* = 78) *			*p* between Responders and Non-Responders	
	Baseline	Post-Intervention	*p* ^a^	Baseline	Post-Intervention	*p* ^a^	Baseline ^a^	Post-Intervention ^a^
Sex (Male/Female)	42/71	-	-	28/50	-	-	-	-
Age (years) ^b^	26.1 ± 6.1	-	-	27.2 ± 6.4	-	-	0.24	-
Weight (kg) ^b^	64.5 ± 9.7	64.6 ± 9.7	0.70	64.2 ± 10.9	64.3±10.6	0.27	0.81	0.85
Height (m) ^b^	164.6 ± 7.9	-	-	164.2 ± 9.1	-	-	0.77	0.71
Body mass index (kg/m^2^) ^b^	23.6 ± 2.7	23.8 ± 2.6	0.23	23.7 ± 2.6	23.8 ± 2.6	0.053	0.84	0.99
Triglycerides (mg/dL) ^b^	110.1 ± 60.7	81.4 ± 41.9	<0.0001	80.3 ± 34.4	111.9 ± 71.9	<0.0001	<0.0001	0.001

^a^ Student *t*-test was used to assess differences pre- vs. post-intervention in responders and non-responders, ^b^ Mean ± standard deviation, * Responders: delta triglycerides pre- vs. post-supplementation <0; non-responders: delta triglycerides ≥0.

**Table 2:** Comparison of allele frequency between French Canadian Caucasians and Mexicans.

Gene	SNP	Minor Allele Frequency		Chi-Squared	*p* Value
		Caucasian	Mexican		
*IQCJ-SCHIP1*	rs12497650	0.32	0.27	2.73	0.10
*IQCJ-SCHIP1*	rs4501157	0.35	0.24	12.57	0.00039
*IQCJ-SCHIP1*	rs13091349	0.17	0.09	9.10	0.0026
*IQCJ-SCHIP1*	rs2044704	0.26	0.41	21.92	2.84 × 10^−6^
*IQCJ-SCHIP1*	rs1962071	0.27	0.38	9.92	0.0016
*IQCJ-SCHIP1*	rs7634829	0.44	0.28	20.77	5.18 × 10^−6^
*IQCJ-SCHIP1*	rs2621294	0.38	0.23	21.57	3.41 × 10^−6^
*IQCJ-SCHIP1*	rs6800211	0.29	0.14	24.59	7.08 × 10^−7^
*IQCJ-SCHIP1*	rs17782879	0.30	0.39	7.12	0.0076
*IQCJ-SCHIP1*	rs1868414	0.33	0.17	25.85	3.69 × 10^−7^
*IQCJ-SCHIP1*	rs2595260	0.25	0.52	61.21	5.12 × 10^−15^
*IQCJ-SCHIP1*	rs6763890	0.34	0.20	18.19	2.00 × 10^−5^
*NXPH1*	rs6956210	0.24	0.13	14.02	0.00018
*NXPH1*	rs2107779	0.55	0.41	16.20	5.70 × 10^−5^
*NXPH1*	rs10273195	0.20	0.24	2.32	0.13
*NXPH1*	rs12216689	0.28	0.32	1.17	0.28
*NXPH1*	rs6963644	0.08	0.04	3.43	0.064
*NXPH1*	rs17150341	0.30	0.16	24.51	7.39 × 10^−7^
*NXPH1*	rs1013868	0.33	0.45	11.91	0.00056
*NXPH1*	rs4318981	0.36	0.36	0.03	0.873
*NXPH1*	rs17153997	0.43	0.30	15.21	9.61 × 10^−5^
*NXPH1*	rs7801099	0.45	0.53	5.60	0.018
*NXPH1*	rs4725120	0.46	0.47	0.16	0.69
*NXPH1*	rs10238726	0.31	0.38	3.61	0.057
*NXPH1*	rs1012960	0.50	0.46	1.61	0.20
*NXPH1*	rs11767429	0.30	0.34	1.25	0.26
*NXPH1*	rs4333500	0.40	0.45	2.03	0.15
*NXPH1*	rs7793115	0.10	0.05	6.60	0.01
*NXPH1*	rs7799856	0.43	0.39	1.10	0.30
*NXPH1*	rs7806226	0.16	0.41	62.40	2.80 × 10^−15^
*NXPH1*	rs13221144	0.23	0.11	22.21	2.45 × 10^−6^
*NXPH1*	rs17406479	0.19	0.31	14.86	0.00012
*NXPH1*	rs10486228	0.18	0.44	61.91	3.59 × 10^−15^
*NXPH1*	rs17154569	0.18	0.08	16.90	3.95 × 10^−5^
*NXPH1*	rs4141002	0.12	0.17	3.69	0.055
*NXPH1*	rs7805772	0.19	0.40	41.06	1.48 × 10^−10^
*NXPH1*	rs2349780	0.38	0.49	10.00	0.0016
*NXPH1*	rs2107474	0.42	0.49	4.03	0.045
*NXPH1*	rs11769942	0.37	0.43	2.98	0.084
*NXPH1*	rs6952383	0.10	0.05	6.26	0.012
*NXPH1*	rs6974252	0.14	0.23	11.04	0.00090
*NXPH1*	rs10265408	0.28	0.31	1.04	0.31
*NXPH1*	rs2189904	0.33	0.18	24.95	5.88 × 10^−7^
*NXPH1*	rs2057862	0.41	0.53	10.99	0.00092
*PHF17*	rs2217023	0.19	0.74	237.40	1.45 × 10^−53^
*PHF17*	rs4975270	0.43	0.47	1.10	0.29
*PHF17*	rs11722830	0.21	0.18	1.16	0.28
*PHF17*	rs12505447	0.19	0.16	1.43	0.23
*PHF17*	rs6534704	0.08	0.03	8.76	0.0031
*PHF17*	rs13148510	0.04	0.01	8.69	0.0032
*PHF17*	rs13143771	0.28	0.32	1.54	0.21
*PHF17*	rs13142964	0.07	0.05	2.43	0.12
*MYB*	rs9321493	0.45	0.45	0.02	0.89
*MYB*	rs11154794	0.13	0.10	1.09	0.30
*MYB*	rs210798	0.42	0.43	0.12	0.73
*MYB*	rs210936	0.48	0.30	27.59	1.50 × 10^−7^
*MYB*	rs7757388	0.16	0.05	26.67	2.42 × 10^−7^
*MYB*	rs17639758	0.03	0.02	1.04	0.31
*MYB*	rs1013891	0.35	0.23	12.98	0.00031
*MYB*	rs2179308	0.51	0.43	4.57	0.033
*IQCJ-SCHIP1*	rs1449009 *	0.29	0.60	77.70	1.20 × 10^−18^
*IQCJ-SCHIP1*	rs61332355 *	0.18	0.33	23.30	1.38 × 10^−6^
*IQCJ-SCHIP1*	rs12485627	0.40	0.32	5.07	0.024
*IQCJ-SCHIP1*	rs2595242	0.52	0.26	53.59	2.47 × 10^−13^
*IQCJ-SCHIP1*	rs7639937	0.25	0.49	46.36	9.84 × 10^−12^
*IQCJ-SCHIP1*	rs9820807	0.16	0.07	17.52	2.84 × 10^−5^
*IQCJ-SCHIP1*	rs1375409	0.29	0.38	7.04	0.0080
*IQCJ-SCHIP1*	rs1967363	0.22	0.36	18.84	1.42 × 10^−5^
*IQCJ-SCHIP1*	rs9824310	0.40	0.46	3.45	0.063
*IQCJ-SCHIP1*	rs11915303	0.27	0.33	3.41	0.065
*IQCJ-SCHIP1*	rs9835214	0.46	0.39	3.79	0.051
*IQCJ-SCHIP1*	rs11921343	0.19	0.26	5.24	0.022
*IQCJ-SCHIP1*	rs13066560	0.16	0.08	13.52	0.00024
*IQCJ-SCHIP1*	rs1675497	0.29	0.32	0.61	0.44
*IQCJ-SCHIP1*	rs9839862	0.11	0.17	5.33	0.021
*IQCJ-SCHIP1*	rs16829875	0.22	0.37	22.29	2.34 × 10^−6^
*IQCJ-SCHIP1*	rs17795566	0.36	0.23	17.17	3.42 × 10^−5^
*IQCJ-SCHIP1*	rs9860588	0.23	0.10	21.25	4.02 × 10^−6^
*IQCJ-SCHIP1*	rs16830408	0.27	0.23	2.17	0.14
*IQCJ-SCHIP1*	rs17798579	0.17	0.16	0.19	0.66
*IQCJ-SCHIP1*	rs2364930	0.40	0.23	25.15	5.31 × 10^−7^
*IQCJ-SCHIP1*	rs9865997	0.14	0.29	25.63	4.14 × 10^−7^
*IQCJ-SCHIP1*	rs2595241	0.26	0.58	86.73	1.25 × 10^−20^
*IQCJ-SCHIP1*	rs7632574	0.19	0.29	10.25	0.0014
*IQCJ-SCHIP1*	rs2621308	0.2589	0.5895	71.53	2.74 × 10^−17^
*SLIT2*	rs2952724	0.3511	0.4789	10.84	0.0010
*PHF17*	rs1216352 *	0.3475	0.5921	38.76	4.80 × 10^−10^
*PHF17*	rs1216365 *	0.6196	0.3421	49.57	1.92 × 10^−12^
*MYB*	rs6920829 *	0.1241	0.1032	0.7113	0.40
*NXPH1*	rs6463808 *	0.1773	0.4	37.86	7.59 × 10^−10^
*NELL1*	rs752088 *	0.3841	0.4579	3.563	0.059

* Single-nucleotide polymorphism (SNP) used in the 7-SNP replicated genetic risk score (GRS).

**Table 3:** Differences in the triglyceride (TG) variation explained by the 5-SNPs GRS in the general linear model with participants retrenched.

Number of Participants Excluded ^a^	Number of Participants Included	% of TG Variance Explained by the GRS	*p*
None	191	11.01	<0.0001
35 responders ^b^	156	12.62	<0.0001
45 responders; 10 non-responders	136	13.20	<0.0001
55 responders; 20 non-responders	116	15.73	<0.0001
65 responders; 30 non-responders	96	17.74	<0.0001
75 responders; 40 non-responders	76	21.30	<0.0001
85 responders; 50 non-responders	56	29.10	<0.0001
95 responders; 60 non-responders	36	28.99	0.0005

^a^ Participants were retrenched in order to reach responders: non-responders ratio of 50:50. ^b^ A 50:50 ratio of responders: non-responders was reached after removing the 35 responders with the lowest delta triglycerides. 5-SNPs were included according to the stepwise regression.

## Supplementary Materials

The following are available online at https://www.mdpi.com/2072-6643/11/4/737/s1, Flowchart of genetic risk score development.
