# Supplementary figures and images for: Genetic Risk Score Predictive of the Plasma Triglyceride Response to an Omega-3 Fatty Acid Supplementation in a Mexican Population
Source: Nutrients. 2019 Mar 29;11(4):737. doi: 10.3390/nu11040737 (PMC6521301; doi:10.3390/nu11040737)

**Supplementary material: Flowchart of genetic risk score development.**

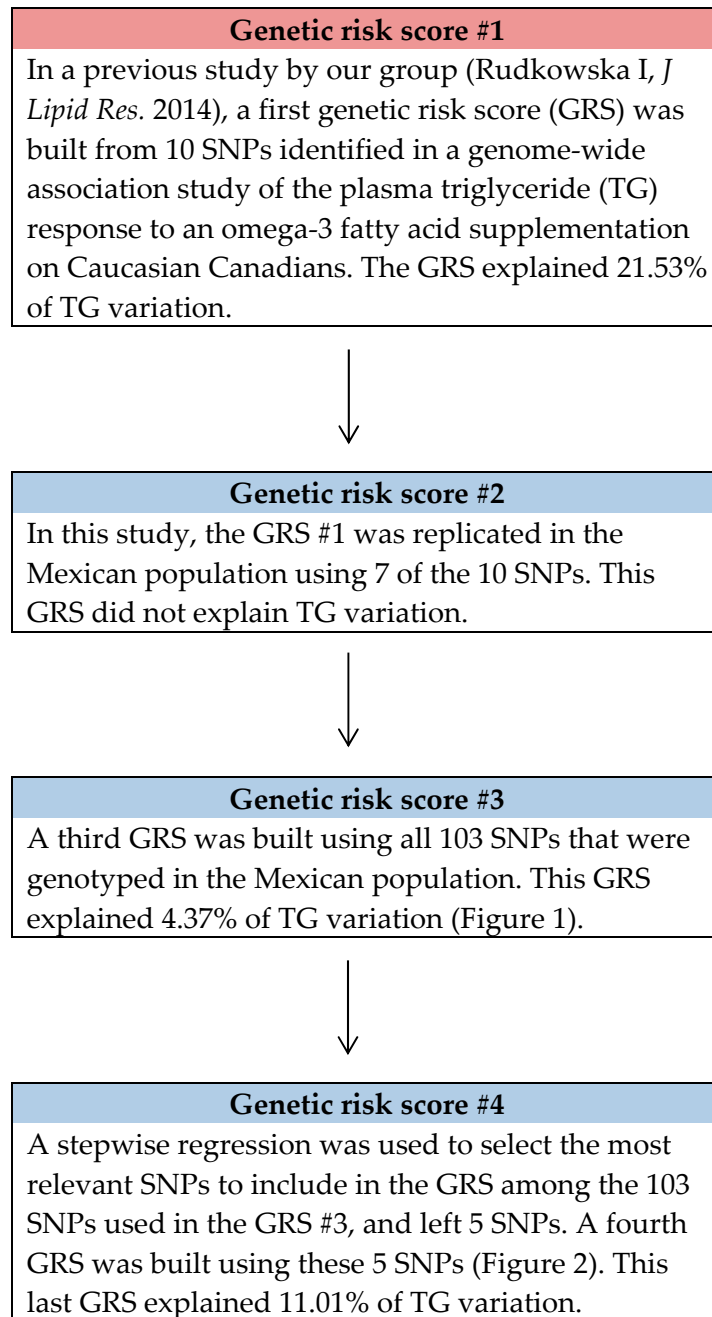

Supplement: Supplementary file 1 [file nutrients-11-00737-s001.pdf]
